# Congenital muscular dystrophy in a dog with a *LAMA2* gene deletion

**DOI:** 10.1111/jvim.16330

**Published:** 2021-12-02

**Authors:** G. Diane Shelton, Katie M. Minor, Stephanie Thomovsky, Ling T. Guo, Steven G. Friedenberg, Jonah N. Cullen, James R. Mickelson

**Affiliations:** ^1^ Department of Pathology, School of Medicine University of California San Diego La Jolla California USA; ^2^ Department of Veterinary and Biomedical Sciences College of Veterinary Medicine, University of Minnesota Saint Paul Minnesota USA; ^3^ Department of Veterinary Clinical Sciences College of Veterinary Medicine, Purdue University West Lafayette Indiana USA; ^4^ Department of Veterinary Clinical Sciences College of Veterinary Medicine, University of Minnesota Saint Paul Minnesota USA

**Keywords:** laminin α2, muscle, myopathy, whole genome sequencing

## Abstract

A 2‐year‐old female spayed dog was presented with a chronic history of short‐strided gait and inability to completely open the jaw. Clinical signs were present since the dog was adopted from a humane society at a few months of age. Serum creatine kinase activity was abnormally high. Neurological examination, electromyography, muscle biopsies with immunofluorescent staining, and whole genome sequencing (WGS) were performed. A dystrophic phenotype was identified histologically in muscle biopsies, deficiency of laminin α2 protein was confirmed by immunofluorescent staining, and a deletion in the *LAMA2* gene was identified by analysis of the WGS data. Congenital muscular dystrophy associated with a disease variant in *LAMA2* was identified.

**Abbreviations**
CK: creatine kinase
CMD: congenital muscular dystrophy
WGS: whole genome sequencing

## 1 INTRODUCTION

Congenital muscular dystrophies (CMDs; muscle disease characteristic of a dystrophic process but not including dystrophies associated with mutations in dystrophin or dystrophin associated proteins) and congenital myopathies (nondystrophic myopathies with characteristic histological and histochemical findings) are the most common groups of congenital onset muscle diseases in humans^1^ and are similarly characterized and classified in veterinary medicine.^2^ Examples of CMDs in humans include but are not limited to myopathies associated with mutations in *COL6* (*COL6A1*, *A2*, and *A3*), *LAMA2* (laminin α2 chain), (*LARGE* and other disorders of hypoglycosylation (α‐dystroglycanopathies), *SEPN1* and *RYR1* related myopathies. In veterinary medicine, specific mutations have been identified in *COL6A3* in Labrador retrievers,^3^ *COL6A1* in Landseer dogs,^4^ and recently in Labrador retrievers with α‐dystroglycanopathy and a mutation within *LARGE1*.^5^

Laminins are large glycoproteins that are structural components of basement membranes in many tissues. In muscle, laminins form the specialized extracellular matrix that immediately abuts and surrounds each muscle fiber.^6^ Laminins are heterotrimeric proteins composed of α, β, and γ subunits that are connected to the sarcolemma via the dystrophin‐glycoprotein complex^6^, ^7^ and integrins.^8^ Multiple isoforms of each type of subunit exist that enable formation of many, sometimes tissue‐specific laminins. The primary heterotrimers present in skeletal muscle are laminin 2 (α2‐β1‐γ1) and laminin 4 (α2‐β2‐γ1) also collectively referred to as merosin. Laminin α2 is also found in the basement membranes of Schwann cells where it is thought to play a role in the ensheathment and myelination of peripheral nerves,^9^ in brain blood vessels,^10^ and in other tissues.

The *LAMA2* gene encodes the α2 subunit of the heterotrimeric laminin‐2 complex, in which predominantly recessive *LAMA2* variants are the basis for a CMD subtype termed the laminin α2‐related muscular dystrophies. Deficiency in laminin α2 protein determined with immunofluorescent staining of muscle cryosections has been reported in humans,^11^ a mouse model,^12^ 3 cats,^13^, ^14^ and in a dog.^15^ Here we describe a disease variant in *LAMA2* in an additional dog resulting in laminin α2‐deficient CMD.

## 2 CASE REPORT: HISTORY AND DIAGNOSTIC TESTING

**FIGURE 1:**
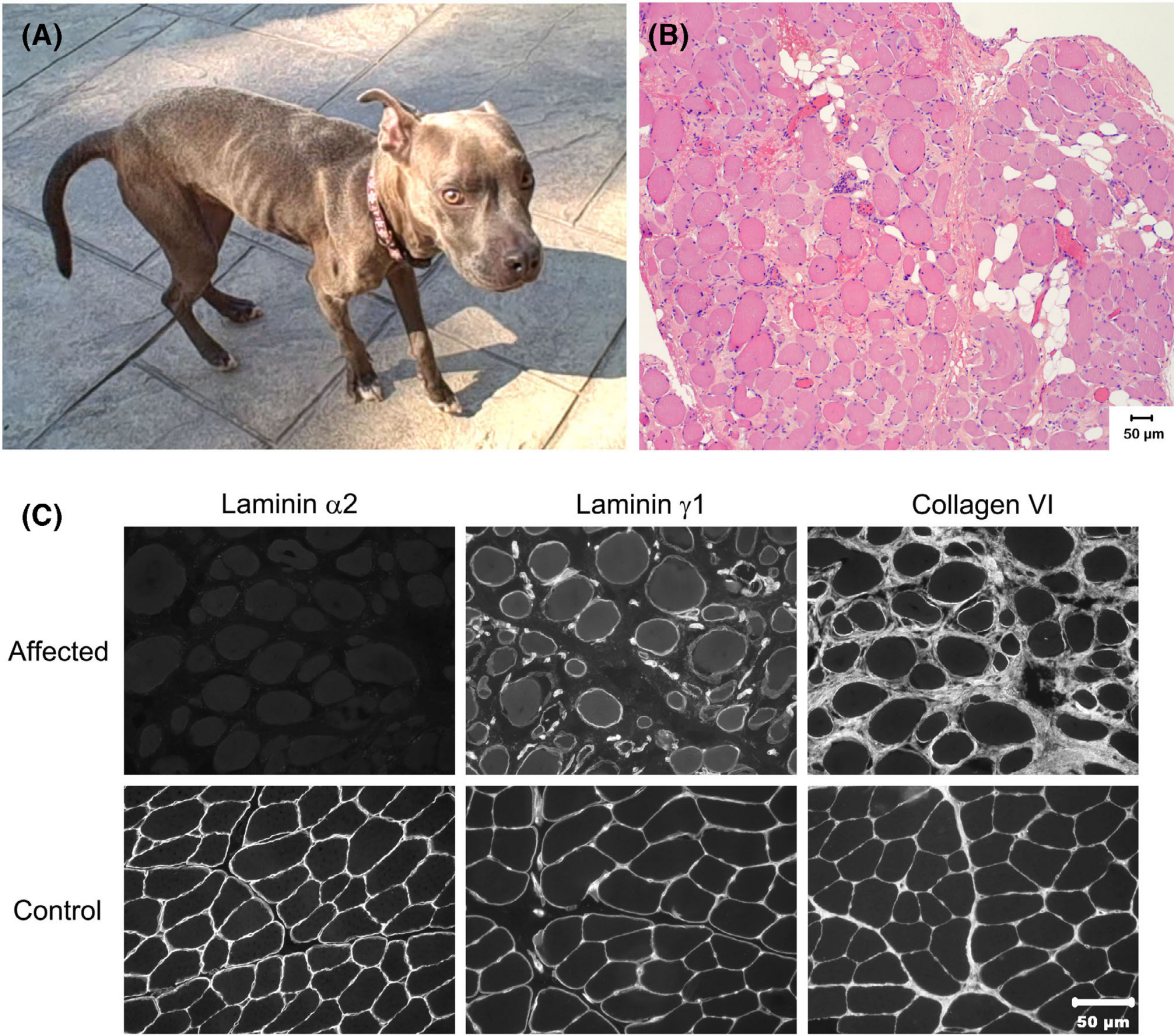
(A) Two‐year‐old female spayed dog with progressive history of muscle atrophy and stiff‐stilted gait. (B) Muscle histopathology showed excessive variability in myofiber size and endomysial fibrosis consistent with a dystrophic phenotype. (C) Immunofluorescent staining of muscle cryosections from the affected dog shows an absence of staining for laminin α2 and normal staining for laminin γ1 and collagen VI. These findings confirm the dystrophy is associated with laminin α2 deficiency

A 2‐year‐old FS Staffordshire terrier (Figure 1A) weighing 11.4 kg was presented to the Purdue University College of Veterinary Medicine with a history of abnormal gait and an inability to open the jaw more than 5 cm. A decreased range of motion at the shoulder joints resulted in a stiff short‐strided gait. Clinical signs were present since adoption from a humane society at a few months of age. Historically the dog had an absent bark when laying in lateral recumbency and the bark was muffled when standing. The owners noted difficulty in righting itself from lateral recumbency. Urination and defecation were normal. Two months before presentation a CBC, serum chemistry panel including creatine kinase (CK) activity, urinalysis, thyroid hormone (T4), and canine trypsin‐like immunoreactivity testing were performed. Important abnormalities included thrombocytosis 504 × 10^3^/μL (170 × 10^3^/μL to 400 × 10^3^/μL), abnormal alanine amino transferase (128 U/L; reference, 10‐118 U/L, aspartate aminotransferase [149 U/L; reference, 15‐66 U/L] and CK 4573 U/L; reference, 59‐895 U/L) activities. Urinalysis, T4, and trypsin‐like immunoreactivity were within normal limits. A lateral thoracic radiograph showed no abnormalities.

Physical examination revealed a pronounced sinus arrhythmia, diffuse muscle atrophy, and mild dental disease. On neurologic examination, the dog had a short‐strided and choppy gait in all 4 limbs, advancing just a few inches with each stride. The dog was tetraparetic without obvious ataxia. When navigating an elevated surface, the dog would hop with both feet together to clear the surface. Mentation and cranial nerves were normal. Hyperesthesia was not detected. Conscious proprioception was decreased in all 4 limbs and both myotatic and flexor withdrawal reflexes were absent in all limbs. Limb sensation was present; limb tone was not recorded. Neurolocalization was to the peripheral nervous system and myopathic or neuropathic diseases were considered more likely than disorders of neuromuscular transmission.

Electrocardiogram and electromyogram followed by muscle biopsies were recommended. A sinus arrhythmia was present on electrocardiography. Electromyogram of the right appendicular and epaxial muscles under general inhalational anesthesia showed diffuse spontaneous activity (fibrillation potentials, positive sharp waves, and complex repetitive discharges) in all muscles evaluated. Unfixed chilled and biopsies immersed in 10% buffered formalin were collected from the latissimus dorsi muscle. The samples were shipped under refrigeration to the Comparative Neuromuscular Laboratory, University of California San Diego.

Histopathology of the latissimus dorsi muscle biopsy showed marked variability in myofiber size, endomysial fibrosis, and sporadic myofibers undergoing necrosis and phagocytosis, consistent with a dystrophic phenotype (Figure 1B). Indirect immunofluorescence staining of muscle cryosections was performed as previously described.^16^ Monoclonal and polyclonal antibodies were used against dystrophy associated proteins including those against the rod (1:20; NCL‐DYS1) and carboxy terminus (1:20; NCL‐DYS2) of dystrophin, α‐sarcoglycan (1:200; affinity purified rabbit polyclonal antibody, gift of Eva Evgvall), β‐sarcoglycan (1:100; NCL‐β‐SARC), and γ‐sarcoglycan (1:100; NCL‐γ‐SARC), laminin α2 (undiluted, 1B4, gift of Eva Engvall),^10^ laminin γ1 (undiluted, 2E8, gift of Eva Engvall), and collagen VI (undiluted, 3G7, gift of Eva Engvall).^17^ Stainings for dystrophin and sarcoglycan localization were similar to control archived muscle. Staining for laminin α2 localization was not detectable in cryosections from the affected dog but normal in control archived muscle (Figure 1C). Staining for other extracellular matrix proteins including laminin γ1 subunit and collagen VI was similar to controls (Figure 1C).

## 3 CASE REPORT: WHOLE GENOME SEQUENCING AND DATA ANALYSIS

**FIGURE 2:**
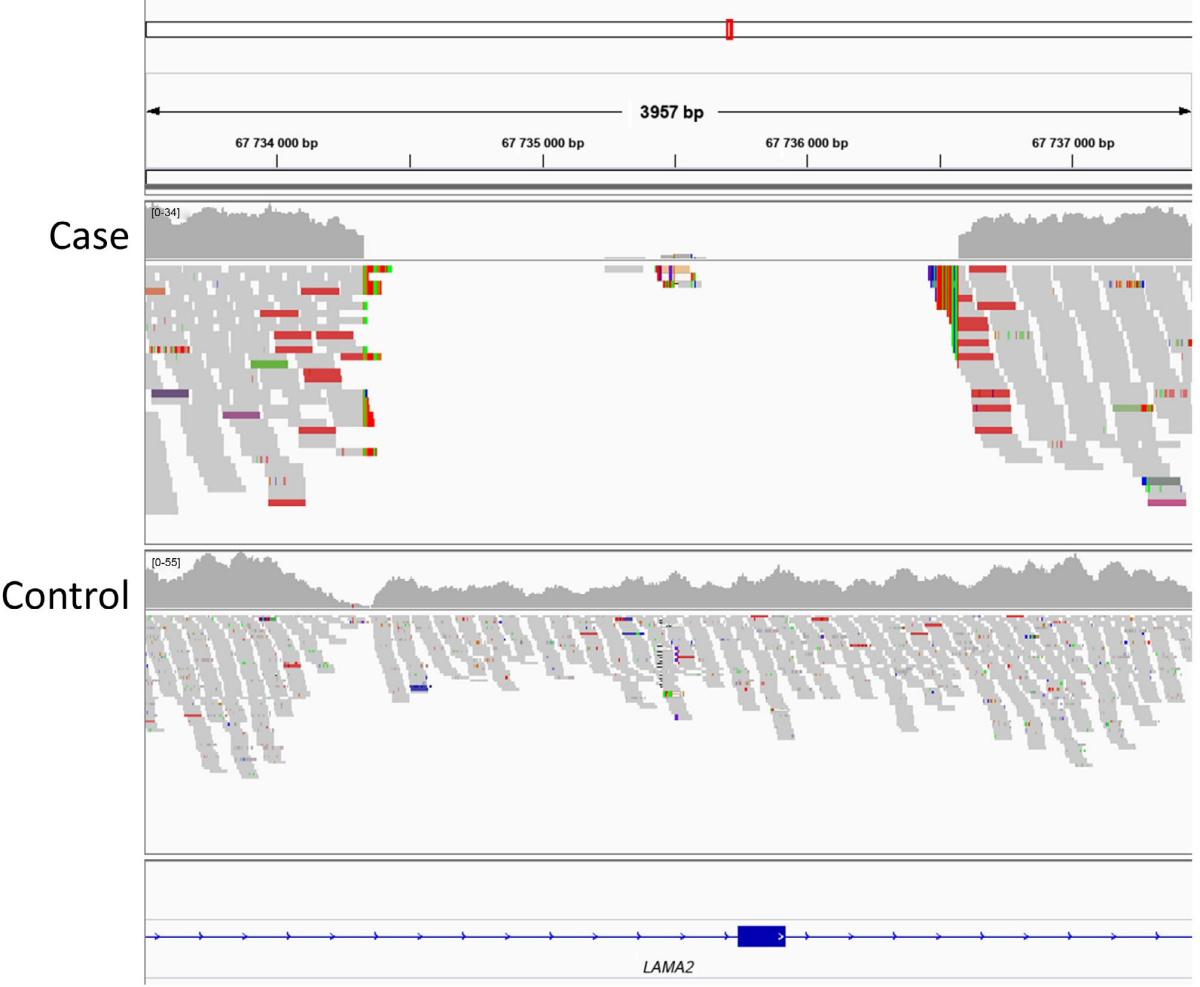
*LAMA2* deletion detected with whole genome sequencing. A 3957 bp segment flanking the 67 735 500 bp position on canine chromosome 1 is shown. Whole genome sequence reads obtained from a normal dog (upper set) and the case (lower set) were aligned to the CanFam3.1 reference genome. Each sequence read that aligns to this region is depicted as a short gray bar (the length of each gray bar is approximately 100 bp) and the sum of reads over any given bp is indicated by the “gray wave” above each dog's set of mapped reads. The absence of any reads aligning to the approximate 67 734 500 to 67 736 500 interval signifies the deletion of that segment in the case dog's genomic DNA. The position of the 180 bp exon 5 in the reference sequence is indicated by the blue box on the bottom

A polymerase chain reaction‐free library was prepared using DNA extracted from muscle of the affected dog and sequenced in 1 lane of an Illumina HiSeq 4000 sequencer by GeneWiz (South Plainfield, New Jersey). The reads were mapped against the dog reference genome assembly (CanFam3.1) as described^18^, ^19^ and are available in NCBI's Short Read Archive at https://www.ncbi.nlm.nih.gov/sra/PRJNA755602. Variants identified in the vicinity of the *LAMA2* gene on chromosome1 in the case were compared to those of control genomes from the University of Minnesota's private whole genome sequencing (WGS) database containing 628 dogs of 60 diverse breeds (including 1 additional American Staffordshire Terrier), but no simple variants unique to the case that altered a codon were found. However, visual analysis of the aligned reads to the reference genome did identify a 2245 bp deletion (chr1:67,734,331‐67,736,575) encompassing *LAMA2* exon 5 (Figure 2). This deletion was observed as an absence of read alignments over this region in the case sequence data, while abundant reads were aligned from the control sequence data.

## 4 DISCUSSION

The *muscularis dystrophia* mouse (*dy/dy*), reported in 1955,^20^ was the first animal model of muscular dystrophy. Deficiency of laminin α2 and a specific mutation was identified in *dy2J/dy2J* mice 40 years later.^12^ Since that time, laminin α2 protein deficiency has been identified in a dog^15^ and in cats^13^, ^14^ but until now a specific mutation in *LAMA2* for cats or dogs has not been reported. Here we describe a deletion encompassing exon 5 of the *LAMA2* gene that is the apparent cause of a laminin α2‐deficient CMD in a dog.

The laminin α2‐related muscular dystrophies in humans are typically autosomal recessive. The dog we report was obtained from a shelter and neither a pedigree nor information on family members was available. However, we speculate that this was also an autosomal recessive condition as, despite abundant flanking coverage, the WGS data had no reads mapping to the region of *LAMA2* exon 5. Additionally, intronic single nucleotide polymorphisms throughout *LAMA2* were all present in the homozygous state. Thus, the case was likely homozygous for the deletion mutation and would have received a mutant allele from each parent, which were presumably heterozygous and phenotypically normal. This scenario also predicts an extremely low mutant allele frequency in dogs in general, as we are not aware of any other cases.

In human patients with CMD, clinical signs of muscular dystrophy and joint contractures are evident at birth or in early infancy with varying degrees of central and peripheral nervous system involvement. The dog of this report presented for clinical evaluation at 2 years of age; however, clinical signs of weakness, stiff gait, and reduced jaw mobility had been present since the dog was adopted from a shelter at a few months of age. The history, neurological examination, increases in CK, electromyography abnormalities, and pathological changes on muscle biopsy were consistent with a chronic and progressive dystrophic myopathy. Immunofluorescence staining confirmed an absence of laminin α2 but no other specific deficiencies of dystrophy associated proteins. Neither cranial nerve abnormalities nor behavioral changes were identified; however, brain magnetic resonance imaging or CT scans were not performed. The decreased range of motion and stiffness in the jaw and shoulder joints of this dog are similar to the limb rigidity and trismus identified in 1 cat previously reported with laminin α2 deficiency.^13^ In both cases, endomysial fibrosis was a prominent histopathologic finding and might have contributed to the muscle stiffness.

Patients with CMD and *dy/dy* mice have reduced motor nerve conduction velocity.^9^, ^21^, ^22^ Evidence of demyelination and Schwann cell abnormalities was described histologically in *dy/dy* mice.^9^, ^22^ In the 2 published feline cases of laminin α2‐deficient CMD,^13^, ^14^ demyelinating neuropathy was identified. In this dog, electrodiagnostic evaluations were limited to needle electromyographic examination and measurements of nerve conduction velocities and compound muscle action potentials not determined. It is possible that the muffled bark and reduced spinal reflexes might have been a result of a demyelinating neuropathy concurrent with the dystrophic myopathy. Without additional electrodiagnostic testing including measurement of the compound muscle action potential amplitude, nerve conduction velocity, and a peripheral nerve biopsy, this is difficult to determine. The limb stiffness could also impair interpretation of the spinal reflexes. Therefore, future studies of *LAMA2* CMDs in dogs and cats should include these electrodiagnostic and tissue evaluations.

In human patients with *LAMA2* related CMD, subclinical cardiac involvement is described, notably right bundle branch block and left ventricular dysfunction, with rare reports of heart failure.^11^ Regular cardiac monitoring with cardiac rhythm assessment by Holter monitoring and cardiac imaging by echocardiogram has been recommended for all *LAMA2* patients. A pronounced sinus arrhythmia was noted in this dog but with no evidence of cardiomyopathy. Cardiomyopathy is relatively common in dystrophin‐deficient muscular dystrophy^23^ but information is limited in the *LAMA2* CMD in dogs.

The canine *LAMA2* gene comprises 65 exons and produces a 9571 bp mRNA that encodes the laminin α2 protein of 3112 amino acids. The deletion reported here encompasses all 180 bp of exon 5 as well as adjacent intron sequences (g.67 734.500_67 736 500del, c.610_789del, p.203_263del). It is possible that deletion of this exon and its flanking splice sites produces a nonfunctional mRNA that would be degraded, prohibiting synthesis of laminin α2 protein and resulting in the absence of immunohistochemical detectable protein. Examination of a database of human *LAMA2* mutations (https://databases.lovd.nl/shared/genes/LAMA2) lists 2127 *LAMA2* variants, in which 1226 are pathogenic or likely pathogenic. Filtering pathogenic or likely pathogenic variants to those within or affecting exon 5 results in 20 variants affecting codons within and limited to exon 5, and 4 variants causing deletion of exon 5 or exons 4‐5. In summary, we believe our identified *LAMA2* variant to be a plausible candidate functional mutation.

The long‐term prognosis for *LAMA2* CMD is poor as the clinical signs are progressive with no specific treatment available. In patients, homozygous loss‐of‐function variants, including large deletions and duplications with complete loss of laminin α2 protein on immunofluorescent staining, are associated with more severe phenotypes.^11^ After diagnosis, this dog was lost to follow‐up and its outcome is not known. In conclusion, this case report expands the spectrum of known mutations associated with CMD in dogs.

## CONFLICT OF INTEREST DECLARATION

Authors declare no conflict of interest.

## OFF‐LABEL ANTIMICROBIAL DECLARATION

Authors declare no off‐label use of antimicrobials.

## INSTITUTIONAL ANIMAL CARE AND USE COMMITTEE (IACUC) OR OTHER APPROVAL DECLARATION

Authors declare no IACUC or other approval was needed.

## HUMAN ETHICS APPROVAL DECLARATION

Authors declare human ethics approval was not needed for this study.
